# The Impact of Role Models, Mentors, and Heroes on Academic and Social Outcomes in Adolescents

**DOI:** 10.7759/cureus.27349

**Published:** 2022-07-27

**Authors:** Hamna Atif, Lindsey Peck, Mary Connolly, Kodi Endres, Leah Musser, Mariam Shalaby, Morgan Lehman, Robert P Olympia

**Affiliations:** 1 Emergency Medicine, Christiana Care Health System, Newark, USA; 2 Obstetrics and Gynecology, Penn State Health Milton S. Hershey Medical Center, Hershey, USA; 3 Internal Medicine, University of Pittsburgh Medical Center, Pittsburgh, USA; 4 Anesthesiology, Penn State Health Milton S. Hershey Medical Center, Hershey, USA; 5 Neurology, Penn State Health Milton S. Hershey Medical Center, Hershey, USA; 6 Psychiatry, Penn State Health Milton S. Hershey Medical Center, Hershey, USA; 7 Internal medicine, Penn State Health Milton S. Hershey Medical Center, Hershey, USA; 8 Emergency Medicine and Pediatrics, Penn State Health Milton S. Hershey Medical Center, Hershey, USA

**Keywords:** social outcomes, academic outcomes, adolescents, heroes, mentors, role models

## Abstract

Background

Identity formation is a dynamic process and key developmental task that begins in adolescence. During this time, children look to adults as role models and mentors. These adults can have a significant impact on adolescents’ decisions of appropriate or inappropriate behaviors, potentially causing a positive or negative change. Little research has been performed to identify these role models and understand how they affect the development of physical and mental health of children.

Objective

The goal of this study is to see if there is a relationship between identified role models, mentors, and/or heroes and adolescents' interest in education, participation in risky behavior, confidence level, happiness, safety, violence-related behaviors, and physical activity.

Methods

In this study, 198 children aged 11-18 years were identified on the scheduling platforms at various Hershey Medical Center sites to take a 10-minute survey via RedCap. The survey identified their role model, mentor, and/or hero and followed up with outcome questions from validated tools.

Results

The results show that 140 participants (70.7%) identified having a role model compared to 88 (44.4%) having mentors and 61 (30.8%) having heroes, and family members were the most identified figures for each category. There were significant differences between identified categories of role models, mentors, and heroes, and interest in education, happiness, risky behavior, and safety, while no significant differences were found for violence-related behavior, physical activity, and confidence level. Adolescents with family heroes had safer behavior (2.39 ± 0.70) than those with celebrity heroes (3.16 ±1.86, p=0.0277), and those with peer heroes (11.3 ± 2.31) had more risky behavior than those with celebrity heroes (9.16 ± 1.98, p=0.0347). However, children with adult peer heroes had a higher interest in education (2.00 ± 0) compared to those with celebrities (3.79 ± 1.03, p=0.0246) or public figures (3.78 ± 1.09, p=0.0333) as their heroes. Additionally, those with family (3.48 ± 1.05) or adult peers (3.32 ± 1.38) as their mentors had a higher interest in education compared to those with same-age peer mentors (5.80 ± 1.30, p=<0.0001). Adolescents with family mentors also had higher happiness scores (3.25 ± 0.33) than those with same-age peer mentors (2.59 ± 1.47, p=0.0358) and also engaged in safer behavior (2.52 ± 0.80) compared to all other categories (3.03 ±1.59, 0.0462).

Conclusion

These results point to the idea that who adolescents choose to look up to has effects on various aspects of their life that could affect both their physical and mental health status, with family members having the most impact. Further research could explore differences between which family members are chosen as role models, mentors, and heroes and what effect they might have on adolescent development.

## Introduction

Identity formation is a dynamic process and key developmental task that begins in adolescence. The American Academy of Child & Adolescent Psychiatry defines a role model as “a person who serves as an example by influencing others” [1]. This influence can have a significant impact on the identity and values that an adolescent adopts. This impact can be positive or negative, depending on the role model that the child identifies with. An association between having a role model with positive outcomes, such as elevated self-esteem, performance in school, and resilience has been established previously [2]. Studies have also shown that having positive role models can protect against engaging in high-risk behaviors, such as participation in violence, sexual intercourse, and substance abuse [3-5]. Just as role models can have a positive influence on adolescent development, role models who participate in socially inappropriate and illegal behaviors can have a negative effect [6]. These “negative role models” have been linked to externalizing behaviors such as violent and nonviolent delinquency, internalizing behaviors such as feelings of anxiety and depression, and substance use behaviors [6]. Parents have been established as influential role models in the lives of adolescents [7]. Having a parent as a role model has been linked to better outcomes in school and less engagement in high-risk behaviors such as substance abuse [8-10]. Having non-familial role models has also been shown to have positive outcomes [11, 12]. Prior research on non-familial role models has been confined to peers and important persons within the community, with little investigation into the effect of celebrities and public figures. There has also been little research done comparing the strength of having familial versus non-familial role models. The current study is unique in that it examines and compares the impact of familial vs. non-familial role models and distinguishes between different categories of non-familial role models such as altruistic figures, celebrities, same-age peers, and adult acquaintances. 

A mentor has been defined as an individual with whom a youth shares a “close, trusting relationship in which the mentor provides guidance and encouragement” [13]. Similar to having a role model, having a mentor has been shown to have a positive impact on adolescents; in particular, performance in school and positive health outcomes has been associated with having a mentor [13-14]. Studies have also suggested that having a mentor decreases the chance of engaging in high-risk behavior, such as substance abuse, violence, smoking, and sexual activity [15]. Just as with role models, there is a lack of research comparing the effect of having a familial versus a non-familial mentor. The current study is distinct in that it directly compares the impact of having a family member as a role model versus a non-family member such as altruistic figures, celebrities, same-age peers, and adult acquaintances. 

Heroes are a category of influencers that have been distinguished as separate from role models and mentors [16], but there is a lack of research indicating the effects of having a hero on adolescent development and outcomes. There has also been little research showing who adolescents are defining as heroes and whether these choices are influencing adolescents in a positive or negative way; this study seeks to contribute to this knowledge by providing a clearer definition of the heroes that adolescents are reporting and comparing the influence of their effects. 

While the importance of role models has been discussed before, little has been investigated in regard to identifying the specific role models, mentors, and heroes that adolescents report. There are two main goals of this study. The first is to identify and define the specific role models, mentors, and heroes that adolescents report. The second goal is to determine if there is a relationship between identified role models, mentors, and/or heroes and interest in education, participation in risky behavior, confidence level, happiness, safety, violence-related behaviors, and physical activity. We achieved this by asking adolescents to name their role models, mentors, and heroes and breaking their responses into separate categories. Each of the categories were then compared among different behaviors to further understand how different role models, mentors, and heroes impact adolescent development.

## Materials and methods

Study design and participants

This cross-sectional study was approved by the Institutional Review Board at the Penn State College of Medicine (Study # 00013970). We prospectively recruited a convenience sample of adolescent patients from one of three Penn State Hershey Medical Center locations (Penn State Pediatric Emergency Department, a general outpatient pediatric, and a Family Medicine Clinic). They participated in an anonymous survey-based study to identify those whom adolescents view as their role models, mentors, and heroes and determine the association of these figures in an adolescent’s life. Participants included in this study were between the ages of 11-18 years and had an accompanying parent or legal guardian present for those under 18 years old. Those whose primary language was not English, children with court-appointed guardians or who are wards of the state, or those who were in severe pain, altered mental status, impaired, or otherwise incapable of providing informed consent as determined by the patient’s physician or study team were excluded from participation. Informed consent was obtained from each participant before the survey was administered. Participants were recruited from July 2019-March 2020 either before or after their appointment visit by a medical student who was a qualified study team member (HA, LP, MC, KE, MS, ML, LM). The participants completed the 10-minute REDCap survey on an iPad or laptop that was provided to them. Participants’ parents and guardians were encouraged not to engage with the participant while they completed the study. Team members were available to answer participant questions at any time during the completion of the survey. There was no follow-up survey or additional contact conducted. All participants were given the option to self-withdraw at any time prior to the completion of the survey. 

Survey and analysis

The survey consisted of various sections including a background/demographics section as well as open-ended responses identifying a role model, mentor, and hero. Additionally, outcome questions, such as overall confidence based on the Rosenberg Self-esteem scale, overall happiness based on the Children’s Happiness Scale, and participation in risky behavior, violence-related behaviors, physical activity, interest in education, and practice of safe behaviors based on the CDC’s National Youth Risk Behavior survey were also addressed (Table 1). A definition of “role model,” “mentor,” and “hero” was provided if the participant indicated that they did not know what those terms meant. A role model was explained as “someone you look up to”. A mentor was explained as “someone in your life that you can go to when you need help/advice?”. A hero was explained as “a person you admire or idolize for their courage, outstanding achievements, or noble qualities”. The full survey can be accessed here: https://redcap.ctsi.psu.edu/surveys/index.php?s=4L973C8FFL.

**Table 1:** Surveys administered

Survey	Link to Survey	Range of Scores	Significance of Result
Interest in Education	https://www.cdc.gov/healthyyouth/data/yrbs/questionnaires.htm	2-9	2 being more interested in education and 9 being less interested
Participation in Risky Behavior	https://www.cdc.gov/healthyyouth/data/yrbs/questionnaires.htm	4-30	4 being less likely to perform risky behavior and 30 being more likely to perform risky behavior
Self-Esteem Scale	https://openpsychometrics.org/tests/RSE.php	0-30	Scores between 15 and 30 suggest higher self-esteem; scores below 15 suggest low self-esteem
Happiness Scale	https://dera.ioe.ac.uk/20502/1/The%20Children's%20Happiness%20Scale.pdf	1.68-4.25	Higher scores indicate higher degree of happiness
The Practice of Safe Behavior	https://www.cdc.gov/healthyyouth/data/yrbs/questionnaires.htm	2-13	2 being more likely to practice safe behavior and 13 being less likely to practice safe behavior
Violence Related Behaviors	https://www.cdc.gov/healthyyouth/data/yrbs/questionnaires.htm	3-18	3 being less likely to participate in violence related behaviors and 18 being more likely
Physical Activity Level	https://www.cdc.gov/healthyyouth/data/yrbs/questionnaires.htm	3-22	3 being more physically active and 22 being less physically active

In this study, influencers are defined as someone whose behaviors or values affect the behavior of the adolescent. Our primary aim was to determine who study participants identified as role models, mentors, and heroes, which are types of influencers. These influencers were further categorized into family members, same-age peers, adult acquaintances, celebrities, and public/altruistic figures. A secondary aim was to determine the association between identified role models, mentors, and heroes and the influence on factors such as interest in education, participation in risky behavior, confidence level, happiness, practicing of safe behaviors, violence-related behaviors, and physical activity level.

For comparative analysis, the identified role models, mentors, and heroes were categorized into the groups: family members, adult acquaintances, same-age peers, celebrities, and public/altruistic figures. For further analysis, the identified role models, mentors, and heroes were categorized into the groups: family members, personal connections (adult acquaintances and same-age peers), and public figures (celebrities and public/altruistic figures).

After data collection, we generated descriptive statistics for continuous variables including means, medians, and standard deviations. Frequencies and percentages were used to describe categorical variables. Statistical significance was determined using two-sample t-tests. All the analysis was carried out on SAS software (SAS Institute Inc. 2013. SAS® 9.4 Statements: Reference. Cary, NC: SAS Institute Inc).

## Results

Analysis was performed on 198 completed surveys. The average participant age was 14 years, with males and females equally represented (Table 2). The majority of participants were white and lived with two parents. 

**Table 2:** Demographics of adolescent participants All values are expressed as no. (%) except where noted. *Value expressed as avg. [SD]

Question	n	Responses	Results
Age (in years)*			
	n=181		14.17 [1.85]
Grade			
	n=197	6	22 (11.17)
		7	30 (15.23)
		8	35 (17.77)
		9	30 (15.23)
		10	28 (14.21)
		11	27 (13.71)
		12	11 (5.58)
		Other	14 (17.11)
Gender			
	n=197	Male	99 (50.25)
		Female	95 (48.22)
		Non-binary (Other)	3 (1.52)
Race			
	n=193	White	135 (69.95)
		Other	22 (11.40)
		Multiple Races	17 (8.81)
		African American	13 (6.74)
		Asian	4 (2.07)
		American Indian/Alaskan Native	2 (1.04)
Household			
	n=195	I live with 2 parents/guardians	142 (72.82)
		I split time between 2 parents/guardians	28 (14.36)
		I live with 1 parent/guardian	25 (12.82)
Reports Having a Role Model			
	n=195	Yes	140 (71.8)
		No	55 (28.2)
Reports Having a Mentor			
	n=193	Yes	88 (45.6)
		No	105 (54.4)
Reports Having a Hero			
	n=192	Yes	67 (34.9)
		No	125 (65.1)

**Figure 1:**
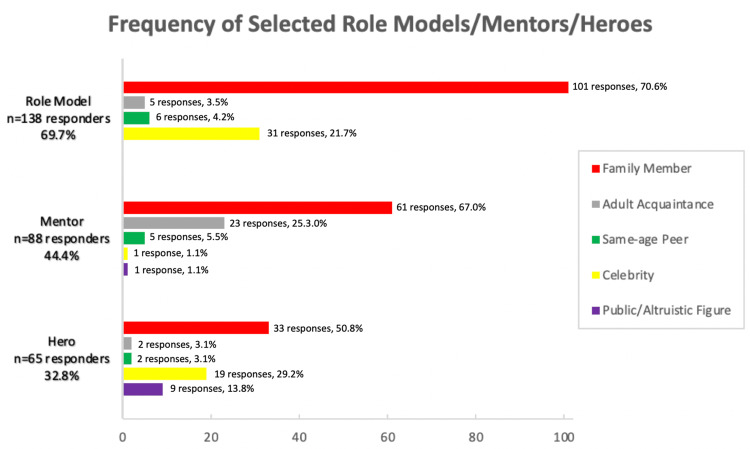
Frequency of selected role models/mentors/heroes by 5 categories Note: Some subjects indicated more than 1 role model/mentor/hero, 2 people did not specify who their role model or hero was.

**Figure 2:**
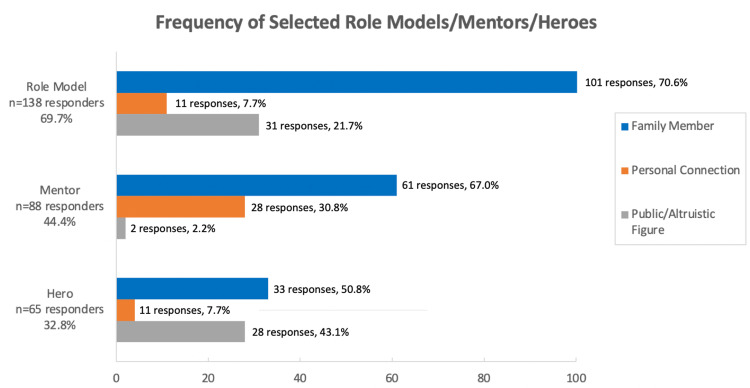
Frequency of selected role models/mentors/heroes by 3 categories Note: Some subjects indicated more than 1 role model/mentor/hero, 2 people did not specify who their role model or hero was.

Overall, the majority of participants identified having a role model (n=140 responders, 70.7%), but fewer participants indicated having a mentor (n=88 responders, 44.4%) or hero (n=67 responders, 33.8%). Across all types of influencers, family members were the most commonly identified, ahead of celebrities, public figures, same-age peers, and adult acquaintances (Figure 1). Additionally, adolescents were more likely to choose someone they do not know personally (public figures or celebrities) compared to someone they have a personal connection to (same-age peers or adult acquaintances) as their hero or role model, but more frequently chose someone they have a personal connection to as a mentor (Figure 2). Statistically significant differences were identified between whether an individual identified a role model, mentor, or hero or not and the measured variables of interest in education, risky behavior, confidence, happiness, and practice of safe behaviors, while no statistically significant differences were found for violence-related behavior or physical activity (Table 3). Of those who selected a role model, mentor, or hero, there were statistically significant differences identified between identified influencer categories and the same variables above, except for confidence being unaffected (Table 4). In general, the data analysis revealed more positive outcome measures for those who chose a family member as their role model, mentor, or hero.

**Table 3:** Behavior differences between having a role model, mentor, or hero and not having one Significant values are listed in bold*

	Behavior	Groups Compared (Group 1 vs. Group 2)	Group 1 mean (SD)	Group 2 mean (SD)	p-value
Role Models	Interest in Education	Yes vs. No	3.71 (1.27)	4.25 (1.24)	0.00082*
	Risky Behavior	Yes vs. No	9.60 (1.71)	10.80 (3.34)	0.0148*
	Confidence Score	Yes vs. No	16.63 (1.97)	15.83 (1.86)	0.0121*
	Happiness Score	Yes vs. No	3.22 (0.40)	2.71 (1.06)	0.0006*
	Participation in Safe Behavior	Yes vs. No	2.70 (1.22)	3.34 (1.99)	0.0291*
	Violent Behavior	Yes vs. No	3.56 (1.47)	3.89 (1.90)	0.2472
	Physical Activity	Yes vs. No	11.66 (4.03)	12.57 (3.71)	0.1477
Mentors	Interest in Education	Yes vs. No	3.60 (1.24)	4.09 (1.28)	0.0071*
	Risky Behavior	Yes vs. No	9.80 (1.80)	10.06 (2.74)	0.4457
	Confidence Score	Yes vs. No	1.95 (16.00)	16.31 (1.98)	0.5123
	Happiness Score	Yes vs. No	3.13 (0.67)	3.02 (0.73)	0.2407
	Participation in Safe Behavior	Yes vs. No	2.69 (1.14)	3.05 (1.73)	0.0873
	Violent Behavior	Yes vs. No	3.63 (1.52)	3.68 (1.69)	0.8167
	Physical Activity	Yes vs. No	11.59 (3.76)	12.20 (4.10)	0.2928
Heroes	Interest in Education	Yes vs. No	3.55 (1.06)	4.03 (1.36)	0.0081*
	Risky Behavior	Yes vs. No	9.77 (1.66)	10.02 (2.62)	0.4286
	Confidence Score	Yes vs. No	16.61 (2.00)	16.29 (1.95)	0.2933
	Happiness Score	Yes vs. No	3.19 (0.33)	3.01 (3.29)	0.0325*
	Participation in Safe Behavior	Yes vs. No	2.60 (1.20)	3.03 (1.62)	0.0380*
	Violent Behavior	Yes vs. No	3.70 (1.75)	3.64 (1.55)	0.8094
	Physical Activity	Yes vs. No	11.28 (3.59)	12.25 (4.10)	0.1097

**Table 4:** Behaviors that had significant differences between identified role models, mentors, or heroes

	Behavior	Groups Compared	Group 1 mean (SD)	Group 2 mean (SD)	p-value
Role Models	Grouping: family, personal connection, public figures				
	Participation in Safe Behavior	Family vs. Public Figures	2.83 (1.36)	2.32 (0.54)	0.0448
Mentors	Grouping: family, adult acquaintances, same-age peers, celebrities, or public figures				
	Interest in Education	Family vs. Same-Age Peers	3.48 (1.05)	5.80 (1.30)	<0.0001
	Interest in Education	Adult Acquaintances vs. Same-Age Peers	3.32 (1.38)	5.80 (1.30)	<0.0001
	Happiness Score	Family vs. Same-Age Peers	3.25 (0.33)	2.59 (1.47)	0.0358
	Grouping: family, personal connection, public figures				
	Happiness Score	Family vs. Personal Connection	3.24 (0.34)	2.91 (0.44)	0.0383
	Participation in Safe Behavior	Family vs. Personal Connection	2.53 (0.80)	3.08 (1.67)	0.0410
Heroes	Grouping: family, adult acquaintances, same-age peers, celebrities, or public figures				
	Interest in Education	Adult Acquaintances vs. Celebrities	2.00 (0.00)	3.79 (1.03)	0.0246
	Interest in Education	Adult Acquaintances vs. Public Figures	2.00 (0.00)	3.78 (1.09)	0.0333
	Risky Behavior	Same-Age Peers vs. Celebrities	12.00 (2.83)	9.16 (1.98)	0.0216
	Participation in Safe Behavior	Family vs. Celebrities	2.39 (0.70)	3.16 (1.86)	0.0277
	Grouping: family, personal connection, public figures				
	Risky Behavior	Personal Connection vs. Public Figures	11.33 (2.31)	9.30 (1.77)	0.0422

Role models

When comparing individuals who identified as having a role model versus those who did not, statistically significant differences were found, all favoring those with a role model. These adolescents had a higher interest in education (p=0.00082), less risky behavior (p=0.0148), higher confidence levels (p=0.0121), higher happiness levels (p=0.0006), and participated in safer behaviors (p=0.0291). There were no statistically significant findings when comparing the role models between five categories: family, same-age peers, adult acquaintances, celebrities, and public figures. However, when comparing who the identified role models were, based on three categories (family member, personal connection, and public figures), those who chose public figures exhibited safer behaviors than those who chose a family member (p=0.0448).

Mentors

When comparing participants who identified as having a mentor versus those who did not, there were significant findings in regard to race and interest in education. Those who identified as white were about equally likely to have a mentor (51%), while their non-white counterparts were less likely to have a mentor (31%, p=0.012). Adolescents that identified as having a mentor had a higher interest in education than those who did not identify a mentor (p=0.007). When comparing the identified mentors based on the five categories (family members, same-age peers, adult acquaintances, celebrities, and public figures), those who identified a family member or adult acquaintance as their mentor had a higher interest in education than those who identified a same-age peer mentor (p<0.0001, p<0.0001). Other grouping comparisons showed no significant findings relating to interest in education. Additionally, adolescents who identified a family member mentor had higher levels of happiness compared to same-age peer mentors (p=0.036). When comparing three categories of mentors (family member, personal connection, and public figure), those with family mentors had higher levels of happiness and participated in safer behaviors than those who identified only a personal connection to their mentor (p=0.039, p=0.041). There were no significant findings between family or personal connection mentors and public figure mentors. 

Heroes

Participants who reported having a hero had significantly more interest in education (p=0.0081), participated in safer behaviors (p=0.0380), and had higher happiness levels (p=0.0325) than those without a hero. When comparing all five categories (family, same-age peer, adult acquaintance, celebrity, public figure), those with adult acquaintance heroes had a higher interest in education than those with celebrity or public figure heroes (p=0.025, p=0.033). Similar to participants with family role models, those who identified family heroes had safer behavior than those who identified celebrity heroes (p=0.0277). Lastly, participants who identified a same-age peer hero participated in riskier behavior than those with celebrity heroes (p=0.022). There were no statistically significant differences when comparing who the identified hero was and happiness. When comparing three categories (family, personal connection, and public figure), participants who had a personal connection to their hero were more likely to have riskier behavior than those who identified a public figure hero (p=0.0422).

## Discussion

Our data support previous research indicating the positive associations of having a role model or mentor on adolescent behaviors and school performance, in addition to family members being most commonly identified as significant persons in adolescent lives [6]. Our study also adds to these associations by providing insight into the correlations between who these identified influencers are and specific behavioral outcomes. We also considered the influences of celebrities/public figures on risky behavior, participation in safe behaviors, and interest in education. Additionally, our findings supplement previous research by analyzing familial vs. non-familial outcome comparisons for role models and mentors, in addition to the associations of having a hero. Curiously, previous research has indicated the correlation between family role models and a decrease in high-risk behaviors [8-10], while our data only showed this association between identifying having a role model or not. Our findings did however reveal a similar association between family role models and an increase in participation in safe behaviors, the only significant finding between an identified role model and our outcome measures.

While our data aligns with previous research indicating the general positive associations of having a mentor on school performance [13, 14], our results also show that family and adult acquaintance mentors specifically have more benefits than peer mentors in regard to interest in education. This could be because children see adults as authority figures and as people who have already successfully obtained an education, unlike school peers. Additionally, children in our study indicated a higher interest in education when they identified having a hero, with more specifically knowing their hero personally, like an adult acquaintance as opposed to public figures or celebrities, having a more significant association. Idolizing someone you know may have more direct positive effects than a person you only know through the media. Decreased interest in education and school disengagement has been shown as a predictor of school dropout, delinquency, official offending, and future substance abuse in adolescence and early adulthood [17]. School dropout specifically is linked to substantially lower income, decreased health, and crime involvement and incarceration. Furthermore, interest in one’s education during adolescence can have drastic impacts on the entirety of one’s life, and obtaining a mentor or hero, ideally, a family member can help decrease the likelihood of these negative outcomes.

Similarly, studies have shown a decrease in risky behaviors when having a positive mentor [15], and our survey responses revealed that having a role model also decreases risky behavior. Interestingly, the participants had significantly decreased risky behaviors when having a celebrity hero versus a peer or friend. This theme remained when comparing levels of risky behavior with individuals who have a hero they know personally to those who do not. However, children with family heroes practiced safer behaviors than those with celebrity heroes, further revealing the potential significant positive impact of family members. Engaging in risky behaviors during adolescence is associated with less favorable outcomes in young adulthood relating to health, economic success, family formation, and incarceration [18]. Often, the earlier an adolescent participates in risky behavior, the more likely they will have negative adult outcomes. This highlights the importance of adolescents finding positive role models, mentors, or heroes in early adolescence.

Adding to previous studies’ associations with having a positive mentor, our data revealed that family mentors were the most significant predictor of happiness and that having a role model also increased happiness. It is well-documented that depression is highly prevalent in adolescence, and is associated with at least one recurrent episode in adulthood [19]. Additionally, adolescent depression has been shown to correlate with lower odds of completing secondary school and higher odds of being unemployed, and lower hourly pay [19]. These associations between adolescent depression and later psychosocial outcomes signify the value of having a positive mentor during adolescence due to its associations with happiness levels.

Surprisingly, our data showed many benefits of having a role model in general, but only had one statistically significant outcome for who this role model was. Our data did not show the known positive associations of family role models with less engagement in high-risk behaviors [8-10]. However, the data revealed that having a role model increases engagement in safer behaviors, with specific family members having more of an association than public figures. We believe this is due to our sample size and that the large majority of participants chose family role models with fewer data to compare to other categories. 

Clinical Implications

Our research has implications for both physicians and parents. Physicians are in a position to teach parents about the importance of role models, mentors, and heroes and emphasize the key role that parents play in behavioral and school-related outcomes for their children. Parents can then use this knowledge and awareness to shape their own behaviors and conversations with adolescents. While we know the positive associations of role models, mentors, and heroes, many adolescents did not identify as having one. Using the tool the authors have created, clinicians and parents can screen their adolescents using the tool “**Look**ing for **help** and **courage**”, by asking the child 3 questions: (1) Is there anyone you look up to? (2) Is there anyone in your life that you can go to for help or advice? and (3) Is there anyone who you admire for their courage, outstanding achievements, or noble qualities? In the case that adolescents answer no to these questions, an opportunity arises to counsel the child on the importance of finding a role model, mentor, or hero, emphasizing the importance of a family member if they are a positive influence.

Limitations

Our study has several limitations, mostly pertaining to the demographics of our sample size. Due to the suburban and somewhat rural location, almost 70% of our study population was white, with almost 73% living with two parents. However, this is only slightly above national data, citing that 69% of adolescents live in families with two parents [20]. These demographics could affect the role models, mentors, and heroes that were identified, and the measured behavioral outcomes could be affected by variables related to these demographics and not solely from their chosen role models, mentors, and heroes. Furthermore, since the family was the most identified category, our results could simply be showing the positive correlations between parental support.

Additionally, our survey lacked a category for Hispanic or Latino participants or those of Middle Eastern or North African descent. Participants of these demographics took part in the study, but unfortunately were not quantitatively accounted for. Their results may or may not have been included in the category “white” or “other.”

Participants were instructed to answer only the questions they felt comfortable answering, so some of the survey questions were not fully completed by our entire study population. As with most surveys, the received responses may not reflect the true behavior of the adolescents, as some of the questions inquired about illegal or risky behaviors.

## Conclusions

Our data demonstrate the powerful positive correlations of family members on the safety, education, and happiness of adolescents aged 11-18. We offer a screening tool to help clinicians and parents identify those without a role model, mentor, or hero with the goal of increasing the number of children with a positive influence. Further research could explore any differences in role models, mentors, and heroes based on race and/or grade point average (GPA). Lastly, additional relevant or more specific adolescent outcomes could be researched than the ones in this study, such as rates of anxiety and depression, teen pregnancy, or obesity.
